# Engineered Strategies for Lipid Droplets-Targeted AIEgens Based on Tetraphenylethene

**DOI:** 10.3390/molecules29245904

**Published:** 2024-12-13

**Authors:** Fei Zhang, Yao Zhang, Zhuoxia Li, Xiaoxiao Wu, Dong Wang, Youling He, Hong Cheng, Baolei Fan, Dan Zhu, Min Li, Ben Zhong Tang

**Affiliations:** 1 Hubei Key Laboratory of Radiation Chemistry and Functional Materials, School of Nuclear Technology and Chemistry & Biology, Hubei University of Science and Technology, Xianning 437000, China; zhangfei@hbust.edu.cn (F.Z.); hongc0529@hbust.edu.cn (H.C.); fanbl_1980@163.com (B.F.); 2 School of Health Service and Management, Shanxi University of Chinese Medicine, 121 University Street, Jinzhong 030619, China; zhangy@sxtcm.edu.cn; 3 Department of Hepatobiliary Surgery, Union Hospital, Tongji Medical College, Huazhong University of Science and Technology, Wuhan 430022, China; m202376170@hust.edu.cn (Z.L.); liminmed@hust.edu.cn (M.L.); 4 Xianning Public Inspection and Testing Center, Xianning 437000, China; wxx963142712@163.com; 5 Center for AIE Research, Shenzhen Key Laboratory of Polymer Science and Technology, Guangdong Research Center for Interfacial Engineering of Functional Materials, College of Materials Science and Engineering, Shenzhen University, Shenzhen 518060, China; wangd@szu.edu.cn; 6 Hubei Key Laboratory of Diabetes and Angiopathy, Xianning Medical College, Hubei University of Science and Technology, Xianning 437000, China; zhudan@hbust.edu.cn; 7 School of Science and Engineering, Shenzhen Institute of Aggregate Science and Technology, The Chinese University of Hong Kong, Shenzhen 518172, China

**Keywords:** aggregation-induced emission, lipid droplets, tetraphenylethene, fluorescence imaging

## Abstract

Lipid droplets (LDs), once regarded as inert fat particles, have been ignored by scientific researchers for a long time. Now, studies have shown that LDs are dynamic organelles used to store neutral lipids in cells and maintain cell stability. The abnormality of intracellular LDs usually causes metabolic disorders in the body, such as obesity, atherosclerosis, diabetes, and cancer, so the LDs have attracted wide attention. The traditional small molecules used for LDs recognition seriously affect the imaging effect due to their poor photo-stability, low signal-to-noise ratios, and aggregation-induced quenching (ACQ). In contrast to ACQ, aggregation-induced emission (AIE) materials, with structural modifiability, can make up for the aforementioned deficiencies in the field of fluorescence imaging and have attracted much attention. In this review, the importance of LDs in vivo, the design principles for LDs recognition, and the recent research progress of AIE compounds with tetraphenylethene (TPE) structure in LDs targets are reviewed. We expect this review to further provide researchers with feasible methods and protocols for expanding LDs identification, imaging, and other applications.

## 1. Introduction

Lipids, a class of hydrophobic molecules in cells, play a very important role in cell metabolism. They are usually involved in the energy source in cells and the formation of cell membranes and serve as precursors for many functional molecules [1,2,3]. However, if the intracellular lipid expression was unusual, this would induce abnormal cell function and even apoptosis, which is called lipid toxicity [4]. Lipid droplets (LDs), the key organelles involved in lipid storage and metabolism, maintain the balance of lipids in cells and meet various needs of cells, and are regarded as the key organelles for energy homeostasis [5]. The abnormal function of LDs can also lead to a variety of diseases, such as obesity, fatty liver, cancer, and so on [6,7].

**Scheme 1:**
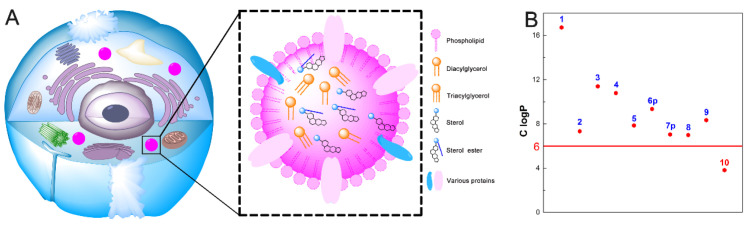
Diagram of LDs and their composition in cells (**A**) and the C logP value (**B**) of TPE-based AIE probe for LDs recognition.

As organelles, LDs have unique structure and are composed of neutral lipids surrounded by a single molecular layer of phospholipids [8,9,10]. Neutral lipids include triacylglycerol and sterol esters. The hydrophilic part of the phospholipid monolayer is towards the cytoplasm, while the hydrophobic part is towards the neutral lipid core, and various functional proteins are distributed on the surface of the phospholipid monolayer (Scheme 1). In addition, LDs are highly motile organelles that maintain interactions with other organelles, such as the nucleus, endoplasmic reticulum, mitochondria, and lysosomes [11,12,13,14]. Despite the awareness of the importance of LDs in cells and the potential dangers of abnormal metabolism, many areas of research in LDs remain unclear. Therefore, by understanding lipid metabolism and observing the morphology and dynamic distribution of LDs, it is very important to study intracellular LDs.

Fluorescence imaging is a non-invasive approach of imaging with high spatial and temporal resolution [15,16]. Due to the rapid development of fluorescence microscopy, researchers have developed auxiliary visualization materials, namely fluorescent probes, which can observe and study various organelles within cells [17,18]. Traditional commercial dyes (BODIPY493/503 or Nile Red) for LDs imaging have the feature of poor photo-stability [19] and low signal-to-noise ratio [20]. Fortunately, a class of LDs-targeted fluorescent probes with good photo-stability and high signal-to-noise ratios have been extensively developed [8,21,22]. Specifically, LDs-targeting fluorescent probes based on tetraphenylethene (TPE) have been extensively developed and can observe the morphology and movement of LDs of cells in real time.

**Scheme 2:**
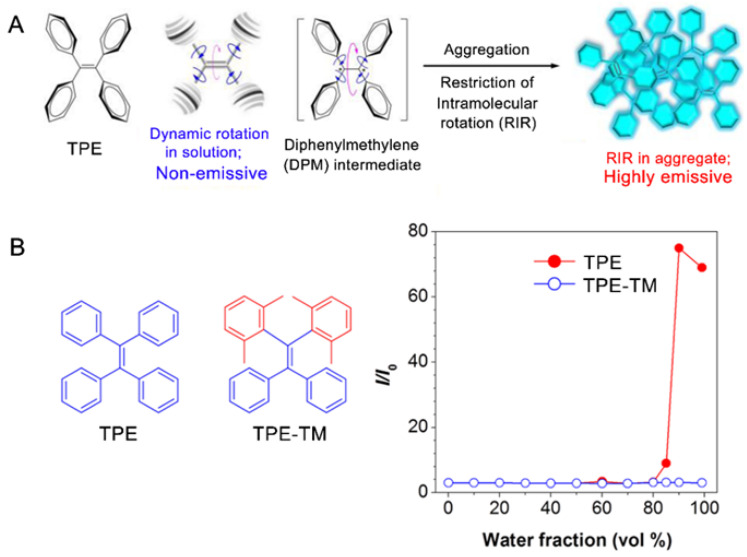
(**A**) Diagram of restriction of intramolecular rotation (RIR) based on TPE. (**B**) Chemical structure of TPE and TPE-TM their plots of I/I_0_ at different water ratios.

Using restriction of intramolecular rotation (RIR) mechanisms, TPE is a classical AIEgen used to build AIE compounds [23,24,25]. In TPE, four benzene rings are connected by a single bond around the center of ethane (Scheme 2). The angle of distortion of four benzene rings and ethene varies greatly in different states. In general, in the dissolved state, the benzene rings can achieve free rotation within the molecule, which is a relaxation channel for the non-radiative decay of the excited state to the ground state. However, in the aggregated state, the free rotation of the phenyl group within the molecule is limited due to physical binding, further blocking the radiationless relaxation channel and opening the radiative decay pathway. In the final form, the fluorescence emission is weak in the solution state and enhances in the aggregation state. However, the opposite phenomenon occurs when multiple methyl groups are modified on the o-positions of the benzene ring connected to double bonds (TPE-TM), directly verifying the RIR mechanism. In contrast to non-AIE dyes, classical AIEgens are known for their low cytotoxicity, high photostability, and large stokes shift. These are exactly the important parameters for dyes to be used for LDs staining in cells. Generally speaking, when hydrophobic small molecules pass through the cell membrane, they can specifically target neutral lipid core through hydrophobic interactions or “like-like” interactions, thus realizing LDs recognition [19]. Organic compounds enter the cell in the presence of the aggregated state and then light up the relevant organelle. From this perspective, AIE compounds containing TPE groups have a unique advantage for LDs recognition and imaging in the cells. Benefiting from the advantages of TPE-based AIE compounds in LDs recognition, this kind of fluorescent probe has been extensively developed and proved to be effective in LDs recognition and visualization in vitro and in vivo.

This review systematically summarizes the progress of TPE-based AIE compounds for LDs recognition or therapy at the cell, tissue, and animal level. Although TPE-based AIE compounds have achieved significant advancements in LDs-specific targeting, studies on imaging and disease models caused by LDs abnormalities have been rarely reported. Therefore, it is of great significance to design and synthesize an LDs probe with excellent specificity and high brightness and further combine it with the disease model caused by LDs abnormality to serve human health. We summarized the design strategies, commonalities, structural characteristics, and applications of LDs-targeted probes, providing researchers with insights in theory, aiming to design high-brightness and highly specific LDs probes for studying the relationship between human health and LDs abnormalities.

## 2. Design Strategies of Fluorescent Probes for LDs Recognition

**Figure 1:**
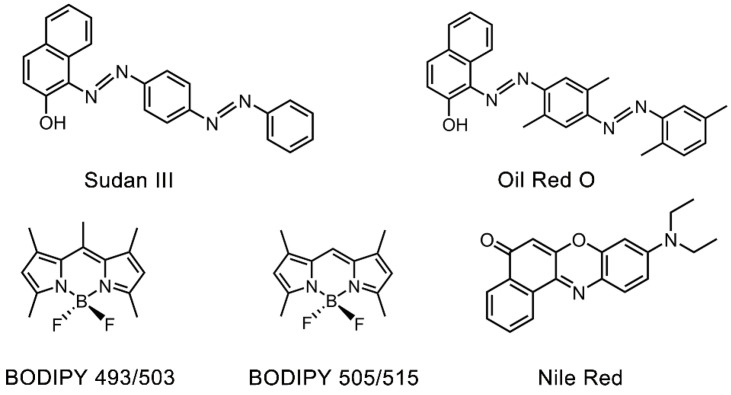
Chemical structure of commonly used commercial LDs dyes.

LDs are composed of neutral lipid cores and are therefore highly hydrophobic. Based on hydrophobic interaction, most of the LDs targeting dyes are highly lipophilic. When hydrophobic small molecules pass through the cell membrane, they can specifically target neutral lipid cores through hydrophobic interactions, thus realizing LDs recognition. At present, commercial LDs-targeting dyes include Diazo dyes (Sudan III [26], Oil Red O [27]), Nile Red [28], BODIPY 493/503, BODIPY 505/515 [29] (Figure 1). These dyes have poor photo stability, poor specificity, and a crosstalk phenomenon caused by small stokes shift. Therefore, the following factors should be considered when designing LDs targeting dyes.

In general, compounds with logP (n-octanol/water partition coefficient) values greater than 5 serve as potential predictors for targeting LDs [30]. The dyes targeting LDs involved in this review have strong hydrophobicity, and C logP values of compounds **1**–**9** are greater than 6. It should be emphasized that the value of C logP of compound **10** is less than 5, and because of this, compound **10** can target both LDs and mitochondria. Secondly, intramolecular hydrogen bonds also affect the distribution of materials in cells. Generally speaking, fewer intramolecular hydrogen bonds in materials can reduce the interaction between materials and water, which is conducive to their distribution in LDs [31]. Finally, due to the presence of neutral lipid cores inside the LDs, the viscosity increases [32,33], which inhibits the rotation of single bonds in the molecule, opening the radiative decay pathway, thereby increasing the radiation transition and further increasing the fluorescence intensity. From the perspective of the luminescence mechanism of TPE, this property of LDs is just enough to target and light up intracellular LDs. Based on this, it is of great advantage to design AIE compounds with TPE configuration for LDs identification. In addition, AIE compounds have a large stokes shift, which is more conducive to biological imaging.

To date, both intramolecular charge transfer (ICT) and AIE fluorescence mechanisms have been exploited to create LD probes. In general, AIE molecules containing triphenylamine (TPA) have strong D-A interaction and are ICT-based for LDs targeting. The solvation effect is greater than the AIE effect in LDs imaging of these molecules. While compounds containing TPE functional groups usually have weaker ICT properties, the principle of light up LDs is mainly based on AIE properties. The AIE effect is greater than the solvation effect in LDs imaging of these molecules [34].

## 3. Recent Advances in LDs Recognition Based on TPE

**Scheme 3:**
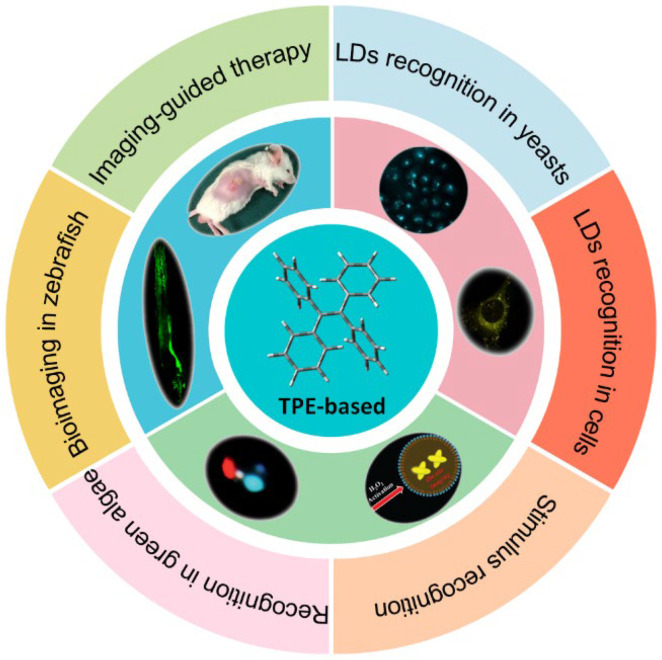
The applications of LDs-targeted TPE-based AIE compounds.

Due to the unique advantages of the TPE group in organelle imaging, we systematically introduced the application of AIE compounds (compounds **1**–**10**) with TPE functional groups in LDs recognition in cellular level, yeasts, algae, zebrafish, and image-mediated tumor therapy (Scheme 3).

### 3.1. Direct Identification at Cellular Level

In this section, direct LDs recognition means that when the compounds enter the cells, they do not interact with the intracellular substances and directly target LDs, thus realizing the recognition of the intracellular LDs (Table 1).

**Figure 2:**
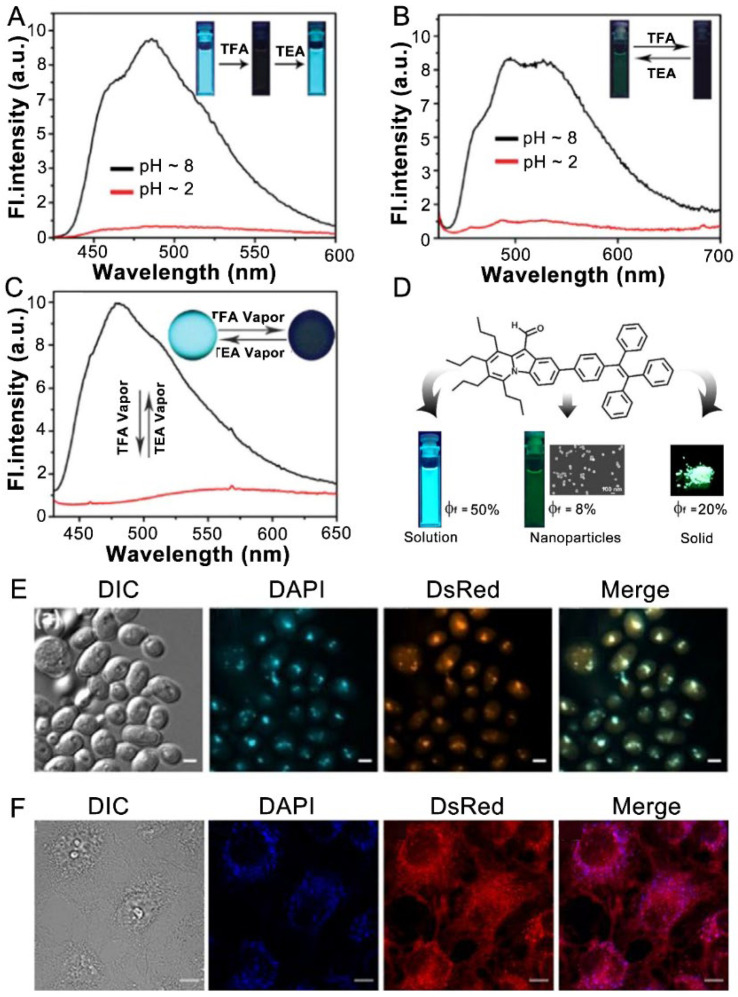
The pH reversibility of compounds in solution (**A**), nanoparticles state (**B**), and solid state (**C**) and their quantum yields (**D**) in different states. Yeast cells (**E**) co-stained with compound **1** and Nile Red (scale bars = 2 μm) (DIC represents differential interference contrast images, DAPI represents fluorescent images with the DAPI filter (blue channel, λ_ex_ = 335–383 nm, λ_em_ = 420–470 nm, compound **1** emission), DsRed represents fluorescent images with the DsRed filter (red channel, λ_ex_ = 538–562 nm, λ_em_ = 570–640 nm, Nile Red emission) and HeLa cells (**F**) co-stained with compound **1** and Nile Red (scale bars = 10 μm) (DIC presents differential interference contrast images, DAPI represents fluorescent images with the DAPI filter (blue channel, λ_ex_ = 405 nm, λ_em_ = 415–585 nm, compound **1** emission), DsRed represents fluorescent images with the tetramethylrhodamine filter (red channel, λ_ex_ = 561 nm, λ_em_ = 570–750 nm, Nile Red emission).

Abhijit Patra [35] prepared compound **1**, a multifunctional fluorescent probe with tetrad alkyl chain substituting pyridoindole based on TPE, which exhibits pH-induced fluorescence switches in solution, nanoparticles, and solid states (Figure 2A–C). By covalently linking TPE functional groups, the compound **1** exhibits strong fluorescence in the aggregated state, further avoiding the fluorescence quenching problem of the compound in the aggregated state and thus developing a compound that exhibits strong fluorescence in both solution and solid state (Figure 2D). Imaging experiments in lower (yeasts) and higher eukaryotes (HeLa cells) demonstrated the specific localization of the compound on LDs (Figure 2E,F). By designing a self-assembled AIE probe with definite physicochemical properties, this study paves the way for the development of a valuable organic fluorescent material with AIE properties for pH sensing and LDs recognition.

**Figure 3:**
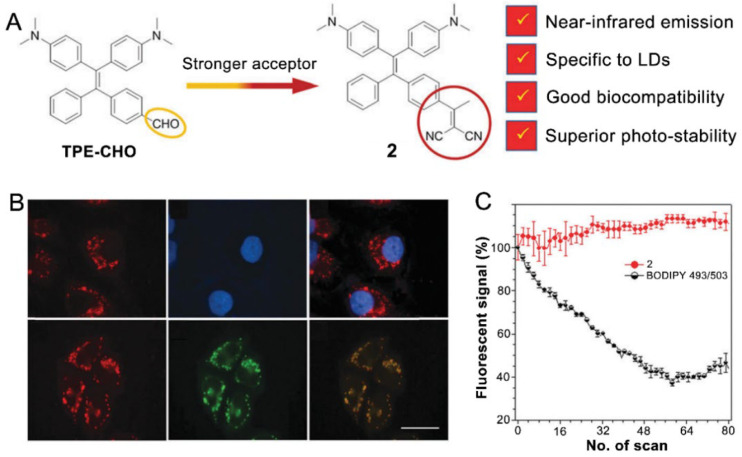
(**A**) Diagram strategy for designing near infrared AIE probe **2**. (**B**) Fluorescence images and overlapping images of HeLa cells co-stained with compound **2** and nuclear targeting dyes DAPI (up), fluorescence images and overlapping images of HeLa cells co-stained with compound **2** and commercial LDs dyes BODIPY 493/503 (down) (scale bars = 30 μm). (**C**) Trend diagram of photostability of compound **2** and commercial LDs dyes BODIPY 493/503.

It is well known that the absorption and emission wavelengths of organic luminescent materials can be effectively regulated by adding electron donors (D) and electron acceptor (A) to the compound. Therefore, by structural modification of TPE-CHO, using dimethylamine as an electron donor (D) and malononitrile as an electron acceptor (A), our research group developed a novel near-infrared AIE LDs probe **2** by increasing the D-A interaction (Figure 3A) [36]. The probe has strong fluorescence emission in the aggregated state. We also demonstrated that probe **2** can specifically target LDs in HeLa cells (Figure 3B), with good biocompatibility and excellent photo-stability (Figure 3C). Compound **2** has a long wavelength of absorption and emission, which provides potential possibilities for LDs recognition in deep tissues, real-time monitoring of LDs in living cells, and provides potential value for early detection and diagnosis of LDs-related diseases.

**Figure 4:**
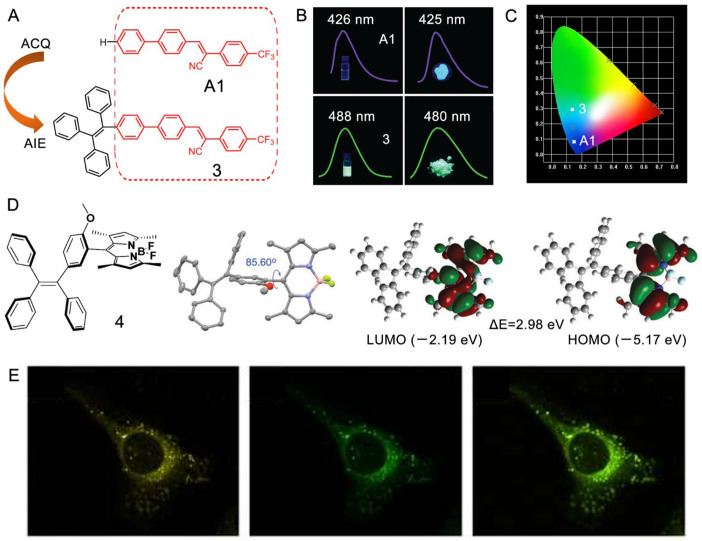
(**A**) Schematic diagram of building AIE compound **3**. (**B**) Emission spectra and photographs of compound **3** in different states. (**C**) CIE 1931 diagram of **A1** and compound **3**. (**D**) The structure of compound **4** and its optimized configuration and molecular orbital diagram. (**E**) Fluorescent images of a HeLa cell incubated with compound **4** (left) and BODIPY (middle) and their superimposed photograph (right).

By adding different rotors to ACQ molecules, a series of compounds with AIE properties, including a TPE-based compound (**3**), have been constructed (Figure 4A) [22]. This strategy not only realizes the transformation from ACQ to AIE, but also achieves the gradual redshift of the emission wavelength, which is nearly 60 nm (Figure 4B), and the corresponding values of CIE diagram are also changed (Figure 4C). Compound **3** has good biocompatibility and can target intracellular LDs. By connecting TPE segments with a BODIPY unit through a conjugated bond, an AIE compound with high torsional angle was designed and synthesized (Figure 4D) [37]. Through theoretical calculation, it is found that the connected phenyl ring of the TPE plane and BODIPY plane are close to the vertical, and the torsion angle reaches 85.60°. The reason for this phenomenon is that the presence of an adjacent methoxy group leads to a large steric resistance. Due to the orthogonal structure of compound **4**, its highest occupied molecular orbital and lowest unoccupied molecular orbital are mainly located in the BODIPY unit rather than the TPE segments, and its energy gaps are 2.98 eV. In biological experiments, it was found that compound 4 can enter cells well. Through the co-localization experiment with commercial LDs-targeted dye BODIPY, it was found that compound **4** can selectively accumulate in LDs in living cells (Figure 4E). This compound has good biocompatibility and high specific LDs recognition and can be used as a tool for LDs-related cell metabolism, providing a new instrument for LDs-related diseases and pharmacological research.

### 3.2. Stimulus Enabled LDs Recognition at Cellular Level

In cellular metabolism, viscosity is a key parameter that determines the metabolic rate, which controls the transport rate of nutrients and the transfer rate of signaling molecules. Indicators of intracellular viscosity are often considered as important parameters for a variety of diseases such as atherosclerosis, diabetes, and Alzheimer’s disease.

**Figure 5:**
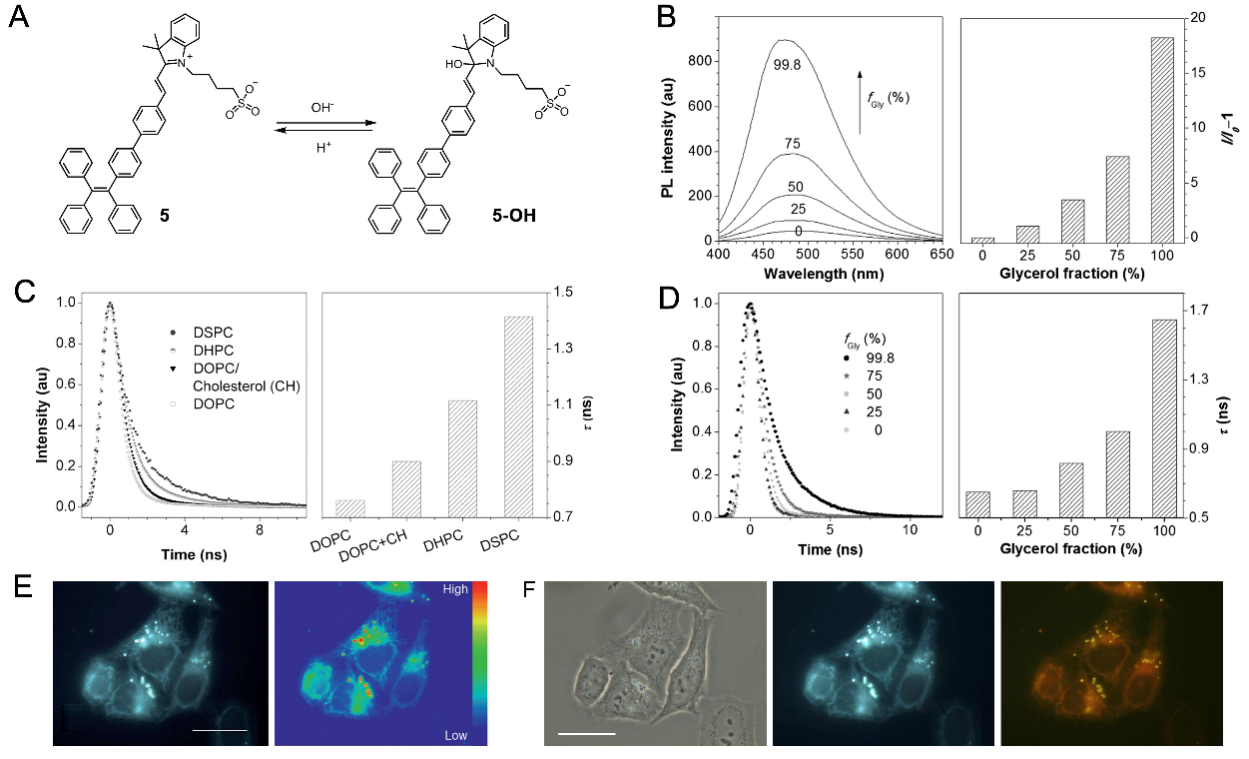
(**A**) The structure of compound **5**, **5-OH** and its pH-sensitive transformation. (**B**) The PL intensity of ethylene glycol mixtures with different glycerol ratios and its plot of I/I_0_ versus glycerol ratios. (**C**) The fluorescence lifetime of **5-OH** in different membrane lipid components. (**D**) The fluorescence lifetime of 5-OH in ethylene glycol mixtures with different glycerol ratios. (**E**) Fluorescent images and surface intensity maps of HeLa cells stained with compound **5** (scale bars = 35 μm). (**F**) Co-localization imaging of HeLa cells stained with **5** and Nile Red (scale bars = 35 μm).

In general, the loose accumulation of lipids in the LDs results in a lower viscosity in this region. However, AIE probes for viscosity detection and LDs identification have not been reported. Therefore, our research group designed and synthesized a TPE-containing luminescent material (compound **5**) with AIE characteristics, which can realize viscosity detection and LDs identification [38]. This compound is a pH-sensitive material, and its fluorescence emission changes from red to blue as the pH of the system increases in solution. Under alkaline conditions, OH^−^ can be used as a nucleophile to react with **5** and further transform into **5-OH** (Figure 5A). In order to investigate the effect of viscosity on the fluorescence intensity of the compound, ethylene glycol mixtures with different glycerol ratios were prepared. As shown in Figure 5B, **5-OH** has weaker fluorescence emission in pure ethylene glycol. With the volume fraction of glycerol increasing, the viscosity of this system gradually increased, and the fluorescence emission intensity of **5-OH** gradually increased, due to the free rotation of the phenyl ring around TPE being limited, resulting in enhanced radiation transition and fluorescence emission. In addition to the improvement of fluorescence intensity, the fluorescence lifetime also gradually extended with the increase of viscosity (Figure 5D). Subsequently, the fluorescence lifetime of **5** under different membrane fluidity was determined (Figure 5C), which further showed that the lifetime of **5-OH** shortened with the increase of fluidity, that is, the decrease of viscosity. Based on the conclusions of the lipid vesicle system model constructed above, we further explore the application of compound **5** in intracellular viscosity sensing. The results of the fluorescence colocalization experiment showed that the blue signal was mainly located in mitochondria, LDs, and other membrane-bound organelles (Figure 5E). In order to further verify its organelle targeting, compound **5** was co-incubated with Nile Red, a commercial LDs dye. The results of co-staining showed that bright spots with strong blue signal in cytoplasm were well co-located with Nile Red, which further indicated that the compound **5** could target LDs well in cells (Figure 5F). This is because the chromogenic part of compound **5** is mainly hydrophobic, causing a large number of dye molecules to gather in the organelle of the lipophilic region. This study provides a new way to screen for drugs that alter intracellular viscosity and could also be applied to other viscosity-altering biological systems.

**Figure 6:**
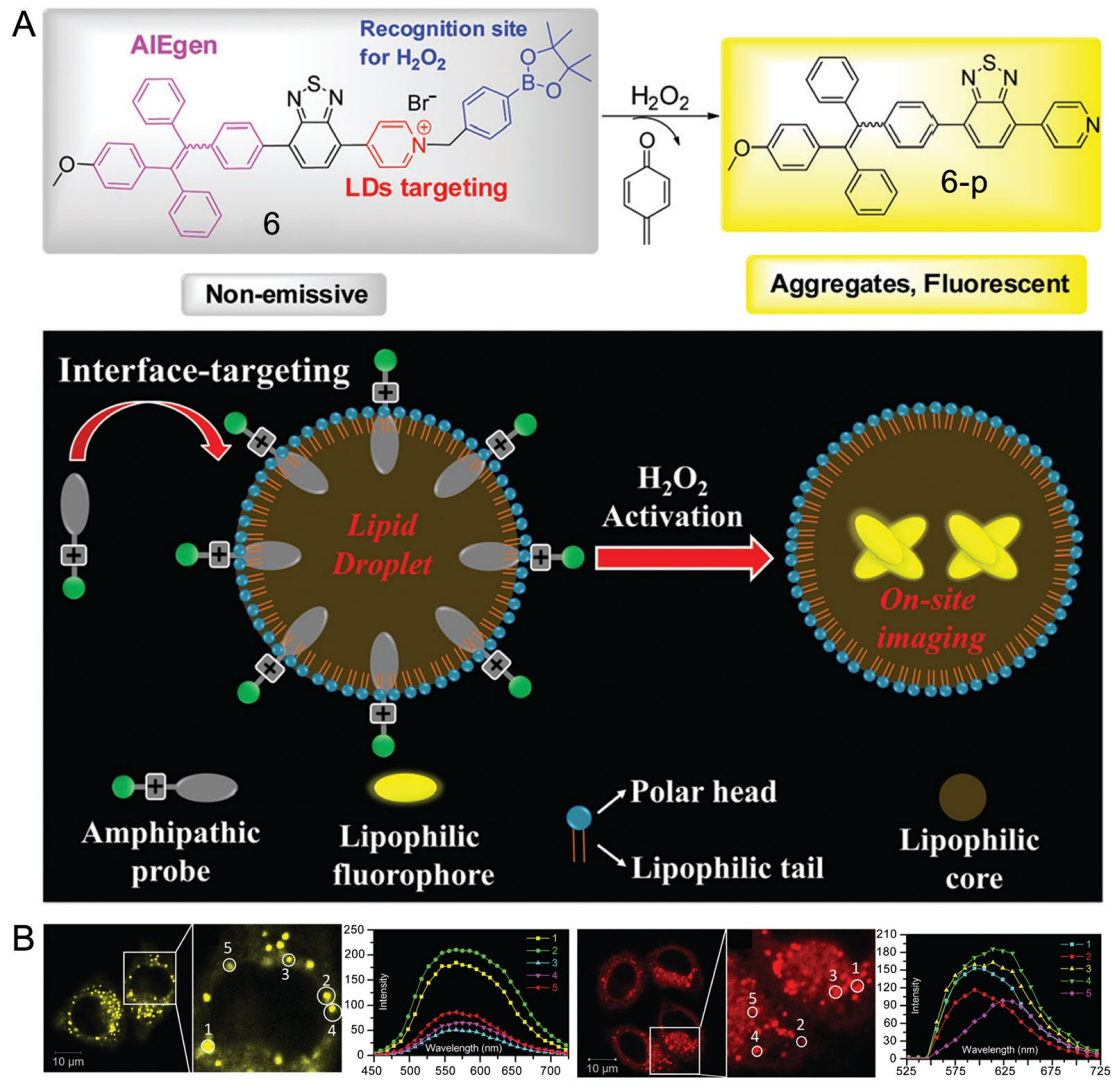
(**A**) Mechanism of H_2_O_2_ recognition by compound **6** and schematic illustration LDs targeting ability. (**B**) Imaging of HeLa cells stained with compound **6** + H_2_O_2_ and Nile Red and their in situ fluorescence spectra.

Unlike other organelles, an LD is an amphiphilic organelle with a special structure, a negatively charged shell, and a lipid core. Therefore, compounds with cationic groups can achieve enrichment on the surface of LDs through interface targeting and electrostatic interaction and thus achieve LDs targeting. Gao’s research group designed a TPE-based AIE probe (**6**), which is a benzothiadiazole-bridged electron compound with donor–acceptor (D-A) interactions [39]. This probe is negatively charged and can target the surface of the LDs. In the presence of H_2_O_2_, the lipophilic compound **6-p** is released by hydrolysis, which forms aggregates in water, emits yellow fluorescence, and then enters LDs, achieving specific recognition of intracellular LDs (Figure 6A). The LDs targeting ability of compound **6** was verified by co-staining with commercial LDs dyes Nile Red. In addition, this compound has a better signal-to-noise ratio than Nile Red, which was verified by the in situ fluorescence spectra in different regions, and showed an ultrahigh selectivity towards LDs (Figure 6B).

**Figure 7:**
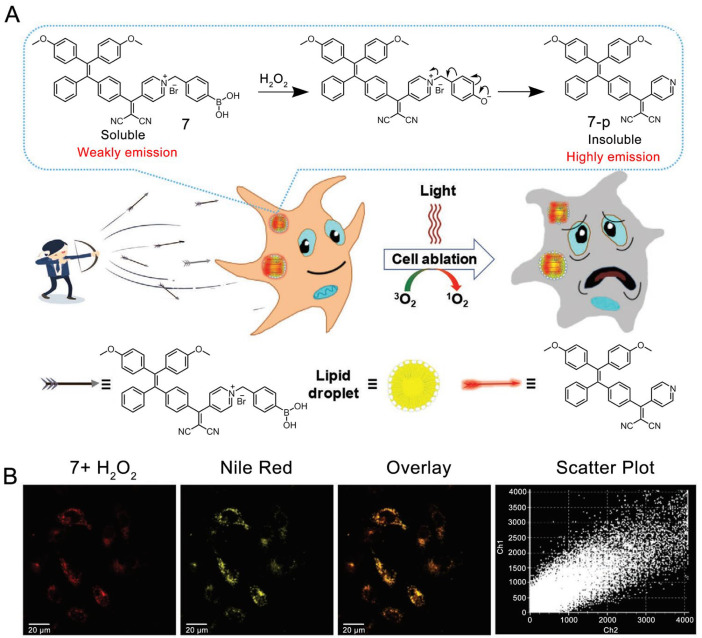
(**A**) Mechanism of H_2_O_2_ recognition by compound **7** and schematic illustration LDs targeting ability and its PDT study. (**B**) Colocalization imaging of MCF-7 cells stained with compound **7** + H_2_O_2_ and Nile Red.

Based on the same strategy, Wang’s team designed another probe (compound **7**) that can recognize endogenous H_2_O_2_ and further light up intracellular LDs [40]. Compound **7** is soluble in water and has weak fluorescence emission due to the presence of pyridinium segment and phenylboronic acid moiety. However, in the presence of H_2_O_2_, the water-soluble compound **7** is converted to the hydrophobic compound **7-p**, which further aggregates in water and emits red fluorescence. In addition, H_2_O_2_-activated compound **7** is a highly efficient photodynamic therapy (PDT) photosensitizer, which can achieve the killing of tumor cells (Figure 7A). The LDs’ targeting ability was demonstrated by exposure to H_2_O_2_ treatment after co-incubation with commercial LDs dyes Nile Red, and it was found to have good overlap, with a Pearson’s colocalization coefficient of 0.89 (Figure 7B).

### 3.3. Fluorescent Images of Green Algae

**Figure 8:**
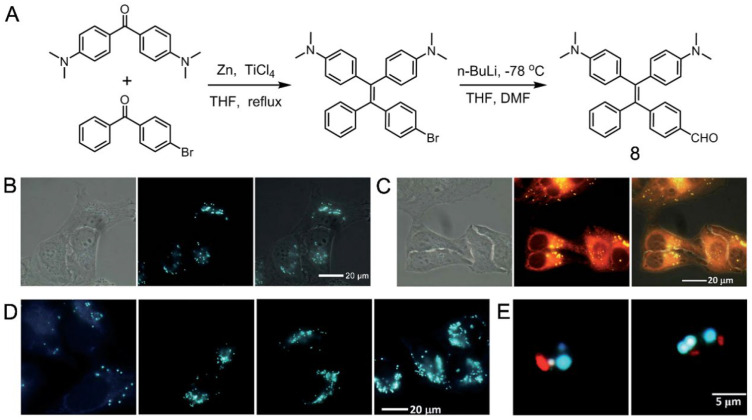
(**A**) Synthetic route of compound **8**. (**B**) HeLa cells incubation with compound **8** (**B**) and Nile Red (**C**) and their corresponding merged images. (**D**) Fluorescent images of HeLa cells incubated with compound **8** after incubation with different contents of oleic acid. (**E**) Fluorescent images of green algae incubated with compound **8** after incubation with different contents of DMSO; the blue emission is from the LDs.

Algae are known to obtain biological matter several times faster than other photosynthetic organisms, so many researchers consider algae a prime candidate to replace gasoline and other fuels. In addition to the speed of growth, algae have many other advantages: they do not require land to grow, and their single-celled nature makes them easier to process into fuel. The content of lipid in algae is one of the most important indicators of its use as fuel. In 2014, our research group prepared a TPE-based AIE LDs dye **8** (Figure 8A) [41]. In cell staining experiments, compound 8 can light up spherical LDs in cells (Figure 8B) and had better signal-to-noise ratios than commercial LDs dyes Nile Red (Figure 8C). Subsequently, the LDs’ targeting ability of cells treated with different concentrations of oleic acid was studied. It was found that with the concentration of oleic acid increasing, more green-blue spots appeared within the cells, further indicating that more LDs were produced in the cells (Figure 8D). Finally, the imaging of LDs in algae was studied by using green algae Nannochloropsis sp. for the model. The experimental results show that the compound can image LDs in algae (Figure 8E). This study can provide a feasible means for screening high bio-fuel contents lipid biofuel (algae).

### 3.4. Bioimaging in Living Cells and Zebrafish

**Figure 9:**
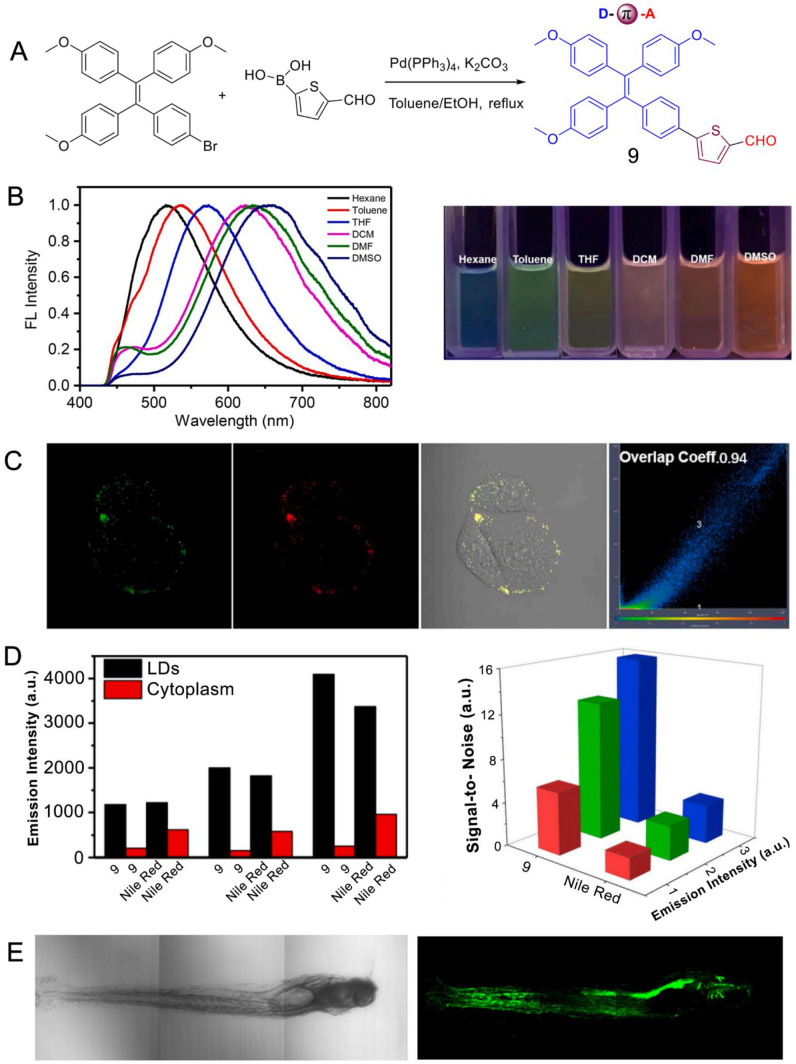
(**A**) Synthesis routes of compound **9**. (**B**) Normalized emission spectra of compound **9** and photographs in different solvents under 365 nm UV light. (**C**) CLSM imaging of HeLa cells co-located by compound **9** and HCS LipidTOX Deep Red at 37 °C. (**D**) The trends’ signal-to-noise ratios of compound **9** and Nile Red stained HeLa cells under different emission intensities. (**E**) Images of bright fields and fluorescent fields of zebrafish incubated with compound **9**.

Cao synthesized a multifunctional AIE compound (**9**) that is sensitive to the environment [42]. The compound is obtained by a simple one-step coupling reaction, and the target compound is a compound with a D-π-A configuration, containing TPE, thiophene, and aldehyde groups (Figure 9A). The compound has an obvious solvation effect at 518~655 nm (Figure 9B), and the powder is sensitive to acid. The fluorescence intensity of the compound gradually decreases at 544 nm under hydrochloric acid vapor atmosphere. Subsequently, under ammonia vapor atmosphere, the fluorescence intensity gradually increased and achieved reversibility. The lipophilic AIE compound **9** can effectively locate intracellular LDs (Figure 9C), realize the specific recognition of LDs in HeLa cells, and achieve the real-time and long-term dynamic tracking of LDs in cells. In addition, this AIE probe has a better signal-to-noise ratio in organelle targeting than commercial LDs probes Nile Red (Figure 9D). Finally, it is also used in imaging experiments of living zebrafish, which further verifies that it is a promising staining material (Figure 9E).

### 3.5. LDs Imaging-Guided Therapy in Mouse

**Figure 10:**
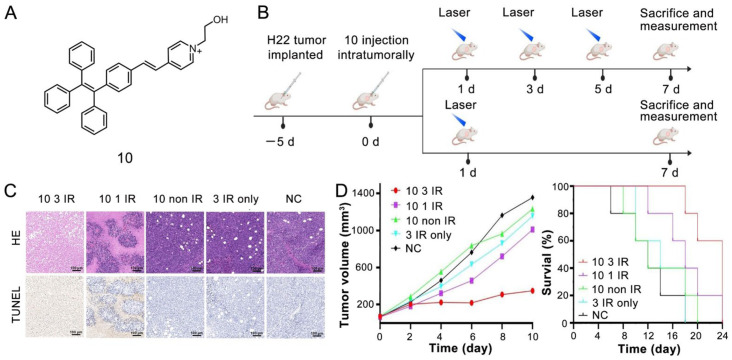
(**A**) Chemical structure of compound **10**. (**B**) Scheme of in vivo experiment. (**C**) H&E and TUNEL staining of slices. (**D**) The volume of tumor and survival rates of mice.

Up to now, PDT has been widely studied in the field of cancer treatment, and great achievements have been made. PDT at the cellular level through recognition of intracellular LDs is relatively common; however, further application to tumor killing in vivo has been rarely reported. Wan’s research group synthesized a TPE-based probe with pyridinium bromide (**10**), which has both hydrophobic structure of TPE and hydrophilic structure of pyridinium bromide (Figure 10A) [43]. Therefore, it can target both intracellular LDs and mitochondria, and this probe has a strong ROS generation ability under light irradiation. The probe has typical AIE properties, showing bright fluorescence emission under aggregates. Moreover, its lipophilic properties make it relatively stable in the aggregation state, and it can achieve long-term retention of LDs in cells (over 7 days). Its strong ROS production ability makes it show better PDT effect in cells. This kind of phenomenon makes it realize long-term tracking in cancer cells and the superior performance of PDT.

Subsequently, the authors studied the tumor treatment effect of this LDs-targeting AIE probe in mice. The therapeutic effect was evaluated by establishing a subcutaneous tumor model (Figure 10B). When the tumor was close to 60 mm^3^, the drug was injected into the tumor. After 12 days of treatment, it was found that the tumor area still had strong fluorescence emission, which further verified that it could achieve long-term retention in the tumor area. Then, the mice were treated with different treatments, single irradiation (1 IR) and three times irradiation (3 IR) after administration, and finally it was found that the effect of 3 IR was significantly better than 1 IR. The H&E staining experiment further verified that the death rate of 3 IR-treated cells was higher than that of the 1 IR group (Figure 10C). Finally, it was found that the tumor volume of the mice treated with 3 IR was significantly smaller than that of the mice treated with 1 IR. In addition, mice in the 3 IR group had a higher survival rate (Figure 10D). This study showed that **10** achieved a prolonged retention of cells and tumors, which in turn achieved long-lasting PDT effects.

## 4. Conclusions and Future Outlook

Due to the modifiability of the structure, simple synthesis operation, large Stokes shift value, excellent photo-stability, and good biocompatibility, AIE compounds show unique advantages in organelle imaging. LDs, as a kind of organelle, have attracted more and more attention because of their close relationship with life and health of humans. This paper reviews the recent progress of TPE-based AIE compounds for LDs recognition imaging in cellular level, yeasts, algae, zebrafish, and image-guided tumor therapy. The general characteristics of AIE compounds for LDs targeting are summarized, which provides valuable guidance for future researchers to design LDs-targeting materials. Although these TPE-based AIE probes perform better than commercial LDs dyes, the mechanism of action of LDs in many biological pathways and physiological processes are not fully understood in cancer cells. In the future, researchers from different fields need to work closely to apply AIE compounds in clinical studies in basic medicine to address the limitations of current biological barriers and synthesize high-performance AIE compounds for further applications to improve cancer treatment efficiency. Once these challenges are addressed, AIEgens-based technologies have a bright future in research and potential clinical applications.

## 5. Challenges and Limitations

Due to its structural modifiability, outstanding biocompatibility, and exceptional photostability, TPE-based AIEgens have been widely used in the field of biological research. This review primarily focuses on the application of TPE-based AIEgens in biological imaging and treatment based on targeting LDs, thereby offering highly meaningful guidance for future research endeavors. Despite the considerable achievements that have been made thus far, there still exist several crucial challenges that need to be addressed in the immediate future.

First and foremost, the emission wavelength of the majority of TPE-based AIE-based fluorescent probes is currently relatively short (less than 600 nm). There is an extremely urgent need to synthesize more highly bright near-infrared AIE fluorescent probes specifically for LDs detection. Obesity’s fundamental characteristic is the elevated levels of LDs. It is of paramount importance to conduct in-depth and comprehensive studies on the disparities in the formation of LDs between obesity-prone individuals and normal individuals’ cells, thereby opening up novel pathways for the utilization of drugs for weight loss.

Secondly, the fluorescence imaging in the second near-infrared region holds numerous advantages such as high resolution, low autofluorescence of tissues, deep penetration depth, and many more, presenting highly promising application prospects. It is essential to design compounds with LDs-targeting capabilities to detect LDs-related diseases Meanwhile, the corresponding equipment for cell-level NIR-II imaging needs to be vigorously promoted and enhanced to fulfill the escalating demand. For the foreseeable future, researchers with backgrounds in physics, biomedicine, and chemistry will work collaboratively to apply AIEgens-based LDs identification to medical and clinical research, which requires a concerted effort and shared expertise to achieve significant breakthroughs and advancements.

Finally, the killing mechanism of PDT and photothermal therapy based on LDs recognition for compounds needs to be further clarified. Compared with other research fields of PDT and photothermal therapy targeting organelles (mitochondria, lysosomes), the research on targeted LDs for therapy, especially in vivo therapy, is relatively rare. In the future, it will be necessary for researchers to clearly explain the cellular damage mechanism of compounds targeting LDs for treatment, so that more neutral molecules targeting LDs for in vitro and in vivo research will explode.

## Figures and Tables

**Table 1:** Photophysical properties and applications of TPE-based LDs probe (n.r. represent unknown).

Dye	λ_ex_/λ_em_, nm	QY	Cell Lines	Reference
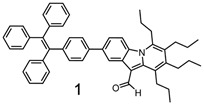	417/490 (THF)	20% (solid state)	yeasts and HeLa cells	[35]
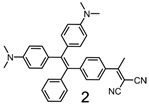	455/724 (THF)	5% (99% water)	HeLa cells	[36]
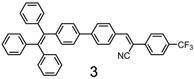	361/488 (water)	n.r.	PC 12 cells	[22]
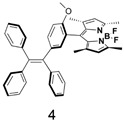	512/515 (THF)	91% (THF)	HeLa cells	[37]
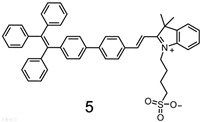	375/480 (99.8% glycerol)	n.r.	HeLa cells	[38]
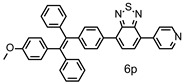	410/590 (THF)	38% (solid state)	HeLa cells	[39]
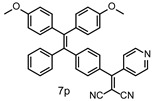	450/625 (PBS)	45.5% (PBS)	MCF-7 cells	[40]
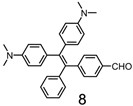	410/525 (THF)	22% (solid state)	HeLa cells	[41]
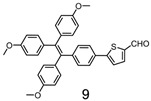	380/655(DMSO)	51% (solid state)	HeLa cells	[42]
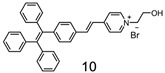	395/650(DMSO)	n.r.	HepG2 cells	[43]

## Data Availability

Dataset available on request from the authors.
